# Supplementary material for: Engagement in types of activities and frequency of alcohol use in a national sample of United States adolescents
Source: PLoS One. 2023 Sep 8;18(9):e0291257. doi: 10.1371/journal.pone.0291257 (PMC10490845; doi:10.1371/journal.pone.0291257)
Supplement: S1 File — (DOCX) [file pone.0291257.s001.docx]

**Supplemental Materials**

| **Supplemental Table S1** | | | | |
| --- | --- | --- | --- | --- |
| *List of items included in measurement models* | | | | |
| Construct | Variable Name | Question Stem | Item Scale | Sample |
| Alcohol Use Frequency | A1 | On how many occasions (if any) have you been drunk or very high from drinking alcoholic beverages…in your lifetime? | 1-7, with higher scores indicating greater alcohol use | Alcohol Subsample |
| Alcohol Use Frequency | A2 | On how many occasions (if any) have you had alcoholic beverages to drink--more than just a few sips… during the last 12 months? | 1-7, with higher scores indicating greater alcohol use | Alcohol Subsample |
| Alcohol Use Frequency | A3 | On how many occasions (if any) have you had alcoholic beverages to drink -- more than just a few sips … during the last 30 days? | 1-7, with higher scores indicating greater alcohol use | Alcohol Subsample |
| Alcohol Use Frequency | A4 | Think back over the LAST TWO WEEKS. How many times have you had five or more drinks in a row? | 1-6, with higher scores indicating greater alcohol use | Alcohol Subsample |
| Enjoyment from School | S1 | Now thinking back over the past year in school, how often did you try to do your best work in school? | 1-5, with higher scores indicating greater reinforcement | Full Sample |
| Enjoyment from School | S2 | Now thinking back over the past year in school, how often did you enjoy being in school? | 1-5, with higher scores indicating greater reinforcement | Full Sample |
| Enjoyment from School | S3* | Now thinking back over the past year in school, how often did you hate being in school? | 1-5, with higher scores indicating greater reinforcement | Full Sample |
| Enjoyment from School | S4 | Now thinking back over the past year in school, how often did you find your school work interesting? | 1-5, with higher scores indicating greater reinforcement | Full Sample |
| Exercise | E1 | How often do you do each of the following? Actively participate in sports, athletics, or exercising | 1-6, with higher scores indicating greater involvement | Full Sample |
| Exercise | E2 | How often do you exercise vigorously (jogging, swimming, calisthenics, or any other active sports)? | 1-6, with higher scores indicating greater involvement | Full Sample |
| Volunteering | V1 | How often do you do each of the following? Participate in community affairs or volunteer work | 1-6, with higher scores indicating greater involvement | Full Sample |
| Employment | J1 | On average over the school year, how many hours per week do you work in a paid job? | 1-8, with higher scores indicating greater involvement | Full Sample |
| Going to concerts | CO1 | How often do you do each of the following? Go to music concerts | 1-6, with higher scores indicating greater involvement | Full Sample |
| Going to the mall | MA1 | How often do you do each of the following? Go to a shopping mall | 1-6, with higher scores indicating greater involvement | Full Sample |
| Media Use | M1 | About how many hours on an average DAY do you spend on social networking sites like Facebook, Twitter, Instagram, etc.? | 1-7, with higher scores indicating greater involvement | Full Sample |
| Media Use | M2 | About how many hours on an average DAY do you spend texting? | 1-7, with higher scores indicating greater involvement | Full Sample |
| Media Use | M3 | About how many hours on an average DAY do you spend playing games on a computer, tv, phone, or other electronic device? | 1-7, with higher scores indicating greater involvement | Full Sample |
| Dating | D1 | On the average, how often (if ever) do you go out with a date? | 1-6, with higher scores indicating greater involvement | Full Sample |
| Alone time | AL1 | How often do you do each of the following? Spend at least an hour of leisure time (free time) alone | 1-6, with higher scores indicating greater involvement | Full Sample |
| Facilitating Activities | C1 | How often do you do each of the following? Get together with friends informally (in your free time) | 1-6, with higher scores indicating greater involvement | Full Sample |
| Facilitating Activities | C2 | How often do you do each of the following? Go to parties or other social affairs | 1-6, with higher scores indicating greater involvement | Full Sample |
| Facilitating Activities | C3 | During a typical week, on how many evenings do you go out for fun and recreation? (Don't count things you do with your parents or other adult relatives) | 1-6, with higher scores indicating greater involvement | Full Sample |
| Facilitating Activities | C4 | How often do your parents (or stepparents or guardians) do the following? Allow you to go out with friends on school nights | 1-4, with higher scores indicating greater involvement | Full Sample |
| Facilitating Activities | C5 | How often do you do each of the following? Go to movies | 1-6, with higher scores indicating greater involvement | Full Sample |
| Facilitating Activities | C6 | How often do you do each of the following? Ride around in a car (or motorcycle) just for fun | 1-6, with higher scores indicating greater involvement | Full Sample |
| Boredom | B1 | I am often bored | 1-5, with higher scores indicating greater boredom | Full Sample |
| Boredom | B2 | I often find myself with nothing to do | 1-5, with higher scores indicating greater boredom | Full Sample |
| Alcohol-involved Activities | AI1 | During the last 12 months, how often (if ever) have you used alcohol in each of the following places? At your home | 1-4, with higher scores indicating greater involvement | Alcohol Subsample |
| Alcohol-involved Activities | AI2 | During the last 12 months, how often (if ever) have you used alcohol in each of the following places? At friends' house | 1-4, with higher scores indicating greater involvement | Alcohol Subsample |
| Alcohol-involved Activities | AI3 | During the last 12 months, how often (if ever) have you used alcohol in each of the following places? At a school dance, a game, or other event | 1-4, with higher scores indicating greater involvement | Alcohol Subsample |
| Alcohol-involved Activities | AI4 | During the last 12 months, how often (if ever) have you used alcohol in each of the following places? At school during the day | 1-4, with higher scores indicating greater involvement | Alcohol Subsample |
| Alcohol-involved Activities | AI5 | During the last 12 months, how often (if ever) have you used alcohol in each of the following places? Near school | 1-4, with higher scores indicating greater involvement | Alcohol Subsample |
| Alcohol-involved Activities | AI6 | During the last 12 months, how often (if ever) have you used alcohol in each of the following places? In a car | 1-4, with higher scores indicating greater involvement | Alcohol Subsample |
| Alcohol-involved Activities | AI7 | During the last 12 months, how often (if ever) have you used alcohol in each of the following places? At a party | 1-4, with higher scores indicating greater involvement | Alcohol Subsample |
| Alcohol-involved Activities | AI8 | During the last 12 months, how often (if ever) have you used alcohol in each of the following places? At a park or beach | 1-4, with higher scores indicating greater involvement | Alcohol Subsample |
| * Indicates item was reverse scored | | |  |  |

| **Supplemental Table S2** | | | |
| --- | --- | --- | --- |
| *Reliability estimates for the Measurement Models* | | | |
| Construct | α | *ω* | Interpretation |
| Enjoyment from School | 0.69 | 0.72 | Acceptable |
| Exercise | 0.80 | 0.80 | Acceptable |
| Media Use | 0.67 | 0.70 | Acceptable |
| Facilitating Activities | 0.68 | 0.69 | Questionable |
| Boredom | 0.87 | 0.87 | Good |
| Alcohol-involved Activities* | 0.83 | 0.82 | Good |
| Alcohol Use* | 0.85 | 0.86 | Good |
| *administered to Alcohol Subsample only | | | |

| **Supplemental Table S3.** *Correlation matrix for No Alcohol Subsample (N=2530)* | | | | | | | | | | |  | | | | | | | | | | | | |
| --- | --- | --- | --- | --- | --- | --- | --- | --- | --- | --- | --- | --- | --- | --- | --- | --- | --- | --- | --- | --- | --- | --- | --- |
|  | C1 | C2 | C3 | C4 | C5 | C6 | S1 | S2 | S3 | S4 | V1 | E1 | E2 | D1 | J1 | CO1 | MA1 | AL1 | B1 | B2 | M1 | M2 | M3 |
| C1 | 1 |  |  |  |  |  |  |  |  |  |  |  |  |  |  |  |  |  |  |  |  |  |  |
| C2 | 0.41 | 1 |  |  |  |  |  |  |  |  |  |  |  |  |  |  |  |  |  |  |  |  |  |
| C3 | 0.40 | 0.33 | 1 |  |  |  |  |  |  |  |  |  |  |  |  |  |  |  |  |  |  |  |  |
| C4 | 0.26 | 0.19 | 0.29 | 1 |  |  |  |  |  |  |  |  |  |  |  |  |  |  |  |  |  |  |  |
| C5 | 0.22 | 0.24 | 0.16 | 0.09 | 1 |  |  |  |  |  |  |  |  |  |  |  |  |  |  |  |  |  |  |
| C6 | 0.34 | 0.29 | 0.29 | 0.17 | 0.17 | 1 |  |  |  |  |  |  |  |  |  |  |  |  |  |  |  |  |  |
| S1 | 0.01 | 0.02 | -0.01 | 0.01 | -0.02 | 0.05 | 1 |  |  |  |  |  |  |  |  |  |  |  |  |  |  |  |  |
| S2 | 0.02 | 0.03 | 0.01 | 0.01 | 0.03 | -0.01 | 0.23 | 1 |  |  |  |  |  |  |  |  |  |  |  |  |  |  |  |
| S3 | 0.04 | 0.05 | 0.01 | 0.02 | 0.04 | -0.01 | 0.18 | 0.62 | 1 |  |  |  |  |  |  |  |  |  |  |  |  |  |  |
| S4 | 0.02 | 0.01 | -0.01 | -0.01 | 0.05 | -0.02 | 0.26 | 0.49 | 0.39 | 1 |  |  |  |  |  |  |  |  |  |  |  |  |  |
| V1 | 0.16 | 0.20 | 0.11 | 0.03 | 0.08 | 0.11 | 0.14 | 0.13 | 0.14 | 0.18 | 1 |  |  |  |  |  |  |  |  |  |  |  |  |
| E1 | 0.25 | 0.21 | 0.25 | 0.09 | 0.11 | 0.14 | 0.10 | 0.14 | 0.12 | 0.09 | 0.24 | 1 |  |  |  |  |  |  |  |  |  |  |  |
| E2 | 0.24 | 0.18 | 0.25 | 0.09 | 0.08 | 0.13 | 0.15 | 0.15 | 0.14 | 0.12 | 0.18 | 0.68 | 1 |  |  |  |  |  |  |  |  |  |  |
| D1 | 0.26 | 0.22 | 0.31 | 0.21 | 0.15 | 0.22 | 0.03 | -0.04 | -0.07 | -0.03 | 0.07 | 0.17 | 0.14 | 1 |  |  |  |  |  |  |  |  |  |
| J1 | 0.05 | 0.06 | 0.07 | 0.06 | 0.03 | 0.15 | -0.01 | -0.02 | -0.02 | 0.02 | 0.03 | 0.02 | 0.02 | 0.12 | 1 |  |  |  |  |  |  |  |  |
| CO1 | 0.13 | 0.25 | 0.14 | 0.11 | 0.29 | 0.18 | 0.01 | 0.04 | 0.06 | 0.06 | 0.15 | 0.08 | 0.06 | 0.14 | 0.04 | 1 |  |  |  |  |  |  |  |
| MA1 | 0.32 | 0.32 | 0.22 | 0.08 | 0.37 | 0.3 | 0.03 | 0.00 | 0.01 | 0.03 | 0.08 | 0.12 | 0.09 | 0.20 | 0.03 | 0.23 | 1 |  |  |  |  |  |  |
| AL1 | 0.05 | 0.05 | 0.04 | 0.04 | 0.01 | 0.03 | -0.04 | -0.05 | -0.08 | -0.05 | -0.03 | -0.03 | -0.08 | -0.01 | -0.06 | -0.02 | 0.01 | 1 |  |  |  |  |  |
| B1 | -0.17 | -0.17 | -0.14 | -0.04 | -0.09 | -0.08 | -0.12 | -0.17 | -0.21 | -0.15 | -0.11 | -0.18 | -0.17 | -0.10 | -0.02 | -0.06 | -0.07 | 0.10 | 1 |  |  |  |  |
| B2 | -0.13 | -0.14 | -0.12 | -0.04 | -0.05 | -0.05 | -0.15 | -0.16 | -0.2 | -0.13 | -0.13 | -0.18 | -0.17 | -0.10 | -0.02 | -0.06 | -0.04 | 0.12 | 0.78 | 1 |  |  |  |
| M1 | 0.12 | 0.10 | 0.12 | 0.03 | 0.07 | 0.2 | -0.02 | -0.13 | -0.16 | -0.09 | -0.08 | -0.01 | -0.03 | 0.13 | 0.05 | 0.04 | 0.23 | 0.04 | 0.12 | 0.13 | 1 |  |  |
| M2 | 0.17 | 0.13 | 0.14 | 0.01 | 0.08 | 0.23 | -0.01 | -0.07 | -0.13 | -0.06 | -0.05 | -0.02 | -0.02 | 0.22 | 0.08 | 0.05 | 0.27 | -0.05 | 0.08 | 0.08 | 0.62 | 1 |  |
| M3 | -0.02 | -0.04 | 0.01 | -0.03 | 0.04 | 0.02 | -0.13 | -0.08 | -0.11 | -0.08 | -0.15 | -0.17 | -0.16 | -0.03 | -0.04 | -0.05 | 0.03 | 0.09 | 0.11 | 0.13 | 0.25 | 0.25 | 1 |
| C = Facilitating Activities, S = Enjoyment from School, V = Volunteering, E = Exercise, D = Dating, J = Employment, CO= Going to concerts, MA = Going to the mall, AL = Time spent alone, B = Boredom, M = Media use | | | | | | | | | | | | | | | | | | | | | | | |

| **Supplemental Table S4.** *Correlation matrix for Alcohol Subsample (N=2096)* | | | | | | | | | | | | | | | | | | | | | | | | | | | | | | | | | | | |
| --- | --- | --- | --- | --- | --- | --- | --- | --- | --- | --- | --- | --- | --- | --- | --- | --- | --- | --- | --- | --- | --- | --- | --- | --- | --- | --- | --- | --- | --- | --- | --- | --- | --- | --- | --- |
|  | C1 | C2 | C3 | C4 | C5 | C6 | S1 | S2 | S3 | S4 | V1 | E1 | E2 | D1 | J1 | CO1 | MA1 | AL1 | B1 | B2 | M1 | M2 | M3 | AI1 | AI2 | AI3 | AI4 | AI5 | AI6 | AI7 | AI8 | A1 | A2 | A3 | A4 |
| C1 | 1 |  |  |  |  |  |  |  |  |  |  |  |  |  |  |  |  |  |  |  |  |  |  |  |  |  |  |  |  |  |  |  |  |  |  |
| C2 | 0.44 | 1 |  |  |  |  |  |  |  |  |  |  |  |  |  |  |  |  |  |  |  |  |  |  |  |  |  |  |  |  |  |  |  |  |  |
| C3 | 0.44 | 0.39 | 1 |  |  |  |  |  |  |  |  |  |  |  |  |  |  |  |  |  |  |  |  |  |  |  |  |  |  |  |  |  |  |  |  |
| C4 | 0.24 | 0.17 | 0.30 | 1 |  |  |  |  |  |  |  |  |  |  |  |  |  |  |  |  |  |  |  |  |  |  |  |  |  |  |  |  |  |  |  |
| C5 | 0.19 | 0.20 | 0.14 | 0.07 | 1 |  |  |  |  |  |  |  |  |  |  |  |  |  |  |  |  |  |  |  |  |  |  |  |  |  |  |  |  |  |  |
| C6 | 0.34 | 0.33 | 0.30 | 0.16 | 0.15 | 1 |  |  |  |  |  |  |  |  |  |  |  |  |  |  |  |  |  |  |  |  |  |  |  |  |  |  |  |  |  |
| S1 | 0.06 | 0.00 | -0.02 | 0.02 | 0.05 | 0.03 | 1 |  |  |  |  |  |  |  |  |  |  |  |  |  |  |  |  |  |  |  |  |  |  |  |  |  |  |  |  |
| S2 | 0.08 | 0.06 | 0.04 | 0.04 | 0.08 | -0.03 | 0.24 | 1 |  |  |  |  |  |  |  |  |  |  |  |  |  |  |  |  |  |  |  |  |  |  |  |  |  |  |  |
| S3 | 0.06 | 0.04 | 0.02 | -0.03 | 0.06 | -0.05 | 0.18 | 0.62 | 1 |  |  |  |  |  |  |  |  |  |  |  |  |  |  |  |  |  |  |  |  |  |  |  |  |  |  |
| S4 | 0.04 | 0.03 | -0.01 | 0.03 | 0.10 | -0.04 | 0.28 | 0.43 | 0.38 | 1 |  |  |  |  |  |  |  |  |  |  |  |  |  |  |  |  |  |  |  |  |  |  |  |  |  |
| V1 | 0.10 | 0.16 | 0.06 | -0.03 | 0.13 | 0.09 | 0.14 | 0.17 | 0.16 | 0.18 | 1 |  |  |  |  |  |  |  |  |  |  |  |  |  |  |  |  |  |  |  |  |  |  |  |  |
| E1 | 0.23 | 0.21 | 0.21 | 0.00 | 0.13 | 0.11 | 0.12 | 0.15 | 0.19 | 0.11 | 0.21 | 1 |  |  |  |  |  |  |  |  |  |  |  |  |  |  |  |  |  |  |  |  |  |  |  |
| E2 | 0.13 | 0.17 | 0.18 | -0.02 | 0.10 | 0.04 | 0.13 | 0.16 | 0.19 | 0.12 | 0.16 | 0.67 | 1 |  |  |  |  |  |  |  |  |  |  |  |  |  |  |  |  |  |  |  |  |  |  |
| D1 | 0.25 | 0.23 | 0.30 | 0.18 | 0.12 | 0.22 | 0.00 | 0.01 | 0.01 | -0.01 | 0.09 | 0.18 | 0.14 | 1 |  |  |  |  |  |  |  |  |  |  |  |  |  |  |  |  |  |  |  |  |  |
| J1 | 0.02 | 0.05 | 0.08 | 0.05 | 0.03 | 0.13 | -0.01 | -0.07 | -0.05 | -0.05 | 0.02 | -0.07 | -0.04 | 0.10 | 1 |  |  |  |  |  |  |  |  |  |  |  |  |  |  |  |  |  |  |  |  |
| CO1 | 0.18 | 0.27 | 0.15 | 0.10 | 0.25 | 0.17 | 0.04 | 0.00 | 0.01 | 0.05 | 0.18 | 0.09 | 0.07 | 0.14 | 0.05 | 1 |  |  |  |  |  |  |  |  |  |  |  |  |  |  |  |  |  |  |  |
| MA1 | 0.29 | 0.28 | 0.18 | 0.10 | 0.29 | 0.25 | 0.09 | 0.08 | 0.04 | 0.07 | 0.11 | 0.09 | 0.06 | 0.16 | 0.03 | 0.22 | 1 |  |  |  |  |  |  |  |  |  |  |  |  |  |  |  |  |  |  |
| AL1 | 0.08 | 0.01 | 0.04 | 0.09 | 0.03 | 0.02 | -0.02 | 0.03 | -0.01 | -0.01 | 0.01 | -0.02 | -0.04 | -0.04 | -0.09 | 0.02 | 0.02 | 1 |  |  |  |  |  |  |  |  |  |  |  |  |  |  |  |  |  |
| B1 | -0.18 | -0.19 | -0.16 | -0.05 | -0.07 | -0.06 | -0.09 | -0.19 | -0.22 | -0.15 | -0.08 | -0.24 | -0.20 | -0.14 | -0.08 | -0.11 | -0.05 | 0.08 | 1 |  |  |  |  |  |  |  |  |  |  |  |  |  |  |  |  |
| B2 | -0.16 | -0.19 | -0.16 | -0.07 | -0.06 | -0.05 | -0.09 | -0.18 | -0.19 | -0.14 | -0.11 | -0.27 | -0.21 | -0.15 | -0.10 | -0.12 | -0.05 | 0.07 | 0.78 | 1 |  |  |  |  |  |  |  |  |  |  |  |  |  |  |  |
| M1 | 0.09 | 0.05 | 0.04 | 0.04 | 0.05 | 0.15 | -0.04 | -0.08 | -0.13 | -0.08 | -0.08 | -0.09 | -0.12 | 0.03 | 0.02 | 0.02 | 0.21 | 0.04 | 0.10 | 0.12 | 1 |  |  |  |  |  |  |  |  |  |  |  |  |  |  |
| M2 | 0.12 | 0.05 | 0.06 | 0.03 | 0.04 | 0.15 | -0.02 | -0.09 | -0.16 | -0.08 | -0.09 | -0.11 | -0.12 | 0.14 | 0.04 | 0.00 | 0.18 | -0.01 | 0.12 | 0.14 | 0.64 | 1 |  |  |  |  |  |  |  |  |  |  |  |  |  |
| M3 | -0.01 | -0.03 | -0.02 | 0.01 | 0.03 | 0.05 | -0.08 | -0.08 | -0.13 | -0.08 | -0.11 | -0.13 | -0.13 | -0.06 | -0.02 | -0.05 | 0.04 | 0.10 | 0.13 | 0.16 | 0.27 | 0.30 | 1 |  |  |  |  |  |  |  |  |  |  |  |  |
| AI1 | 0.01 | 0.08 | 0.09 | 0.04 | -0.01 | 0.06 | -0.10 | -0.06 | -0.07 | -0.03 | 0.01 | -0.02 | 0.01 | 0.05 | 0.05 | 0.05 | -0.01 | 0.04 | 0.08 | 0.07 | 0.02 | 0.03 | 0.00 | 1 |  |  |  |  |  |  |  |  |  |  |  |
| AI2 | 0.21 | 0.30 | 0.27 | 0.10 | 0.01 | 0.18 | -0.08 | -0.06 | -0.09 | -0.09 | 0.02 | 0.11 | 0.08 | 0.12 | 0.01 | 0.14 | 0.06 | 0.06 | -0.03 | -0.05 | 0.09 | 0.08 | -0.03 | 0.33 | 1 |  |  |  |  |  |  |  |  |  |  |
| AI3 | 0.12 | 0.24 | 0.14 | 0.06 | 0.04 | 0.12 | -0.07 | -0.04 | -0.06 | -0.03 | 0.01 | 0.04 | 0.06 | 0.11 | 0.02 | 0.13 | 0.12 | 0.04 | 0.02 | 0.01 | 0.12 | 0.08 | 0.05 | 0.25 | 0.40 | 1 |  |  |  |  |  |  |  |  |  |
| AI4 | 0.02 | 0.09 | 0.09 | 0.00 | -0.01 | 0.03 | -0.17 | -0.11 | -0.08 | -0.06 | -0.03 | -0.07 | -0.04 | 0.08 | 0.00 | 0.05 | 0.01 | 0.03 | 0.05 | 0.07 | 0.03 | 0.08 | 0.05 | 0.24 | 0.27 | 0.42 | 1 |  |  |  |  |  |  |  |  |
| AI5 | 0.08 | 0.17 | 0.13 | 0.03 | 0.01 | 0.09 | -0.18 | -0.10 | -0.09 | -0.06 | -0.01 | -0.01 | 0.01 | 0.12 | 0.01 | 0.09 | 0.03 | 0.00 | 0.03 | 0.04 | 0.06 | 0.09 | 0.04 | 0.27 | 0.34 | 0.52 | 0.68 | 1 |  |  |  |  |  |  |  |
| AI6 | 0.10 | 0.22 | 0.18 | 0.07 | -0.01 | 0.17 | -0.13 | -0.11 | -0.13 | -0.08 | -0.04 | -0.02 | -0.01 | 0.08 | 0.07 | 0.09 | 0.08 | 0.02 | 0.02 | 0.02 | 0.13 | 0.13 | 0.06 | 0.30 | 0.49 | 0.54 | 0.47 | 0.57 | 1 |  |  |  |  |  |  |
| AI7 | 0.21 | 0.38 | 0.28 | 0.09 | 0.03 | 0.19 | -0.11 | -0.04 | -0.07 | -0.07 | 0.02 | 0.11 | 0.12 | 0.17 | 0.03 | 0.17 | 0.12 | -0.01 | -0.05 | -0.06 | 0.10 | 0.09 | 0.02 | 0.30 | 0.68 | 0.43 | 0.22 | 0.33 | 0.52 | 1 |  |  |  |  |  |
| AI8 | 0.12 | 0.22 | 0.16 | 0.05 | 0.00 | 0.11 | -0.15 | -0.08 | -0.10 | -0.06 | 0.01 | 0.04 | 0.05 | 0.11 | 0.00 | 0.11 | 0.04 | -0.01 | -0.02 | 0.00 | 0.06 | 0.05 | 0.00 | 0.27 | 0.42 | 0.42 | 0.34 | 0.46 | 0.50 | 0.51 | 1 |  |  |  |  |
| A1 | 0.16 | 0.30 | 0.24 | 0.11 | -0.03 | 0.17 | -0.17 | -0.09 | -0.12 | -0.10 | -0.01 | 0.04 | 0.01 | 0.14 | 0.08 | 0.13 | 0.02 | -0.02 | -0.04 | -0.03 | 0.08 | 0.06 | 0.02 | 0.34 | 0.55 | 0.36 | 0.25 | 0.35 | 0.47 | 0.54 | 0.44 | 1 |  |  |  |
| A2 | 0.18 | 0.33 | 0.23 | 0.10 | 0.00 | 0.15 | -0.15 | -0.08 | -0.09 | -0.08 | -0.01 | 0.10 | 0.06 | 0.12 | 0.06 | 0.12 | 0.05 | 0.01 | -0.07 | -0.07 | 0.08 | 0.06 | 0.00 | 0.44 | 0.57 | 0.36 | 0.24 | 0.32 | 0.44 | 0.58 | 0.43 | 0.68 | 1 |  |  |
| A3 | 0.14 | 0.29 | 0.20 | 0.07 | -0.01 | 0.15 | -0.15 | -0.09 | -0.09 | -0.08 | -0.02 | 0.04 | 0.01 | 0.09 | 0.08 | 0.13 | 0.06 | -0.06 | -0.07 | -0.05 | 0.07 | 0.07 | 0.02 | 0.31 | 0.42 | 0.31 | 0.22 | 0.33 | 0.42 | 0.47 | 0.37 | 0.57 | 0.74 | 1 |  |
| A4 | 0.13 | 0.27 | 0.20 | 0.03 | 0.00 | 0.14 | -0.14 | -0.09 | -0.09 | -0.07 | -0.04 | 0.03 | 0.03 | 0.09 | 0.08 | 0.12 | 0.04 | -0.07 | -0.06 | -0.04 | 0.06 | 0.08 | 0.05 | 0.26 | 0.39 | 0.35 | 0.19 | 0.35 | 0.40 | 0.42 | 0.35 | 0.51 | 0.58 | 0.74 | 1 |
| C = Facilitating Activities, S = Enjoyment from School, V = Volunteering, E = Exercise, D = Dating, J = Employment, CO= Going to concerts, MA = Going to the mall, AL = Time spent alone, B = Boredom, M = Media use, AI = Alcohol-involved activities, A = Alcohol use frequency | | | | | | | | | | | | | | | | | | | | | | | | | | | | | | | | | | | |

| **Supplemental Table S5** | | | | | | |
| --- | --- | --- | --- | --- | --- | --- |
| *Summary of results for Full Sample Measurement Model (Model 1)* | | | | | | |
|  |  | *Unstandardized B* | *S.E.* | *z* | *p* | *Standardized β* |
| Latent Variables: | |  |  |  |  |  |
| Enjoyment from School | |  |  |  |  |  |
|  | S1 | 0.29 | 0.02 | 19.69 | < .001 | 0.32 |
|  | S2 | 0.84 | 0.02 | 56.15 | < .001 | 0.82 |
|  | S3 | 0.81 | 0.02 | 50.87 | < .001 | 0.74 |
|  | S4 | 0.55 | 0.02 | 37.39 | < .001 | 0.57 |
| Exercise | |  |  |  |  |  |
|  | E1 | 1.55 | 0.03 | 48.04 | < .001 | 0.89 |
|  | E2 | 1.25 | 0.03 | 42.35 | < .001 | 0.76 |
| Volunteer | |  |  |  |  |  |
|  | V1 | 1.09 | 0.01 | 95.75 | < .001 | 1.00 |
| Employment | |  |  |  |  |  |
|  | J1 | 1.49 | 0.02 | 95.87 | < .001 | 1.00 |
| Going to Concerts | |  |  |  |  |  |
|  | CO1 | 0.65 | 0.01 | 95.85 | < .001 | 1.00 |
| Going to the Mall | |  |  |  |  |  |
|  | MA1 | 0.90 | 0.01 | 95.79 | < .001 | 1.00 |
| Media Use | |  |  |  |  |  |
|  | M1 | 1.24 | 0.03 | 46.04 | < .001 | 0.77 |
|  | M2 | 1.36 | 0.03 | 48.93 | < .001 | 0.83 |
|  | M3 | 0.54 | 0.03 | 20.44 | < .001 | 0.33 |
| Dating | |  |  |  |  |  |
|  | D1 | 1.28 | 0.01 | 94.39 | < .001 | 1.00 |
| Alone Time | |  |  |  |  |  |
|  | AL1 | 1.22 | 0.01 | 95.87 | < .001 | 1.00 |
| Facilitating Activities | |  |  |  |  |  |
|  | C1 | 0.82 | 0.02 | 45.02 | < .001 | 0.67 |
|  | C2 | 0.68 | 0.02 | 42.22 | < .001 | 0.63 |
|  | C3 | 0.87 | 0.02 | 39.48 | < .001 | 0.61 |
|  | C4 | 0.37 | 0.02 | 19.71 | < .001 | 0.36 |
|  | C5 | 0.25 | 0.01 | 21.85 | < .001 | 0.36 |
|  | C6 | 0.88 | 0.03 | 34.17 | < .001 | 0.53 |
| Boredom | |  |  |  |  |  |
|  | B1* | 1.18 | 0.02 | 78.36 | < .001 | 0.90 |
|  | B2* | 1.18 | 0.02 | 78.36 | < .001 | 0.86 |
| Covariances: | |  |  |  |  |  |
| Enjoyment from School | |  |  |  |  |  |
|  | Exercise | 0.22 | 0.02 | 12.34 | < .001 | 0.22 |
|  | Volunteer | 0.21 | 0.02 | 13.32 | < .001 | 0.21 |
|  | Employment | -0.06 | 0.02 | -3.55 | < .001 | -0.06 |
|  | Concerts | 0.03 | 0.02 | 1.90 | 0.06 | 0.03 |
|  | Mall | 0.05 | 0.02 | 2.78 | 0.01 | 0.05 |
|  | Media Use | -0.20 | 0.02 | -10.92 | < .001 | -0.20 |
|  | Dating | -0.06 | 0.02 | -3.32 | < .001 | -0.06 |
|  | Alone Time | -0.05 | 0.02 | -2.86 | < .001 | -0.05 |
|  | Facilitating Activities | 0.02 | 0.02 | 1.01 | 0.31 | 0.02 |
|  | Boredom | -0.27 | 0.02 | -15.47 | < .001 | -0.27 |
| Exercise | |  |  |  |  |  |
|  | Volunteer | 0.25 | 0.02 | 16.26 | < .001 | 0.25 |
|  | Employment | -0.02 | 0.02 | -1.49 | 0.14 | -0.02 |
|  | Concerts | 0.09 | 0.02 | 5.61 | < .001 | 0.09 |
|  | Mall | 0.12 | 0.02 | 7.26 | < .001 | 0.12 |
|  | Media Use | -0.10 | 0.02 | -5.28 | < .001 | -0.10 |
|  | Dating | 0.19 | 0.02 | 12.02 | < .001 | 0.19 |
|  | Alone Time | -0.04 | 0.02 | -2.25 | 0.02 | -0.04 |
|  | Facilitating Activities | 0.37 | 0.02 | 21.13 | < .001 | 0.37 |
|  | Boredom | -0.27 | 0.02 | -15.25 | < .001 | -0.27 |
| Volunteer | |  |  |  |  |  |
|  | Employment | 0.03 | 0.02 | 1.73 | 0.08 | 0.03 |
|  | Concerts | 0.17 | 0.01 | 11.40 | < .001 | 0.17 |
|  | Mall | 0.10 | 0.02 | 6.58 | < .001 | 0.10 |
|  | Media Use | -0.10 | 0.02 | -6.30 | < .001 | -0.10 |
|  | Dating | 0.07 | 0.02 | 4.80 | < .001 | 0.07 |
|  | Alone Time | -0.01 | 0.02 | -0.80 | 0.42 | -0.01 |
|  | Facilitating Activities | 0.20 | 0.02 | 11.74 | < .001 | 0.20 |
|  | Boredom | -0.12 | 0.02 | -7.49 | < .001 | -0.12 |
| Employment | |  |  |  |  |  |
|  | Concerts | 0.05 | 0.02 | 3.37 | < .001 | 0.05 |
|  | Mall | 0.03 | 0.02 | 2.24 | 0.03 | 0.03 |
|  | Media Use | 0.07 | 0.02 | 4.28 | < .001 | 0.07 |
|  | Dating | 0.12 | 0.02 | 8.16 | < .001 | 0.12 |
|  | Alone Time | -0.07 | 0.02 | -4.83 | < .001 | -0.07 |
|  | Facilitating Activities | 0.13 | 0.02 | 7.83 | < .001 | 0.13 |
|  | Boredom | -0.05 | 0.02 | -3.18 | < .001 | -0.05 |
| Concerts | |  |  |  |  |  |
|  | Mall | 0.23 | 0.01 | 16.19 | < .001 | 0.23 |
|  | Media Use | 0.05 | 0.02 | 2.92 | < .001 | 0.05 |
|  | Dating | 0.16 | 0.02 | 10.52 | < .001 | 0.16 |
|  | Alone Time | 0.00 | 0.02 | 0.23 | 0.82 | 0.00 |
|  | Facilitating Activities | 0.34 | 0.02 | 21.42 | < .001 | 0.34 |
|  | Boredom | -0.09 | 0.02 | -5.18 | < .001 | -0.09 |
| Mall | |  |  |  |  |  |
|  | Media Use | 0.28 | 0.02 | 17.81 | < .001 | 0.28 |
|  | Dating | 0.18 | 0.02 | 12.39 | < .001 | 0.18 |
|  | Alone Time | 0.00 | 0.02 | 0.28 | 0.78 | 0.00 |
|  | Facilitating Activities | 0.46 | 0.01 | 31.92 | < .001 | 0.46 |
|  | Boredom | -0.06 | 0.02 | -3.75 | < .001 | -0.06 |
| Media Use | |  |  |  |  |  |
|  | Dating | 0.20 | 0.02 | 11.98 | < .001 | 0.20 |
|  | Alone Time | 0.02 | 0.02 | 0.96 | 0.34 | 0.02 |
|  | Facilitating Activities | 0.28 | 0.02 | 14.91 | < .001 | 0.28 |
|  | Boredom | 0.17 | 0.02 | 9.14 | < .001 | 0.17 |
| Dating | |  |  |  |  |  |
|  | Alone Time | -0.01 | 0.02 | -0.75 | 0.46 | -0.01 |
|  | Facilitating Activities | 0.45 | 0.02 | 30.52 | < .001 | 0.45 |
|  | Boredom | -0.12 | 0.02 | -7.25 | < .001 | -0.12 |
| Alone Time | |  |  |  |  |  |
|  | Facilitating Activities | 0.08 | 0.02 | 4.72 | < .001 | 0.08 |
|  | Boredom | 0.11 | 0.02 | 6.40 | < .001 | 0.11 |
| Facilitating Activities | |  |  |  |  |  |
|  | Boredom | -0.23 | 0.02 | -12.12 | < .001 | -0.23 |
| Intercepts: |  |  |  |  |  |  |
|  | S1 | 4.14 | 0.01 | 306.20 | < .001 | 4.51 |
|  | S2 | 3.04 | 0.02 | 200.42 | < .001 | 2.95 |
|  | S3 | 2.79 | 0.02 | 174.24 | < .001 | 2.57 |
|  | S4 | 2.69 | 0.01 | 188.35 | < .001 | 2.78 |
|  | E1 | 4.25 | 0.03 | 164.61 | < .001 | 2.43 |
|  | E2 | 4.00 | 0.03 | 159.58 | < .001 | 2.44 |
|  | V1 | 2.26 | 0.02 | 141.21 | < .001 | 2.08 |
|  | J1 | 1.72 | 0.02 | 78.67 | < .001 | 1.16 |
|  | CO1 | 1.50 | 0.01 | 157.26 | < .001 | 2.32 |
|  | CM1 | 2.76 | 0.01 | 208.97 | < .001 | 3.08 |
|  | M1 | 3.85 | 0.02 | 162.14 | < .001 | 2.39 |
|  | M2 | 3.59 | 0.02 | 148.67 | < .001 | 2.19 |
|  | M3 | 3.70 | 0.02 | 152.85 | < .001 | 2.25 |
|  | D1 | 1.90 | 0.02 | 99.52 | < .001 | 1.49 |
|  | AL1 | 5.02 | 0.02 | 279.05 | < .001 | 4.12 |
|  | C1 | 4.00 | 0.02 | 221.42 | < .001 | 3.26 |
|  | C2 | 2.73 | 0.02 | 172.80 | < .001 | 2.55 |
|  | C3 | 2.47 | 0.02 | 116.06 | < .001 | 1.73 |
|  | C4 | 2.65 | 0.02 | 155.76 | < .001 | 2.59 |
|  | C5 | 2.46 | 0.01 | 239.48 | < .001 | 3.53 |
|  | C6 | 3.39 | 0.02 | 139.03 | < .001 | 2.05 |
|  | B1 | 3.38 | 0.02 | 166.33 | < .001 | 2.59 |
|  | B2 | 3.19 | 0.02 | 149.76 | < .001 | 2.34 |
|  | Enjoyment from School | 0.00 | 0.00 | 0.00 |  |  |
|  | Exercise | 0.00 | 0.00 | 0.00 |  |  |
|  | Volunteer | 0.00 | 0.00 | 0.00 |  |  |
|  | Employment | 0.00 | 0.00 | 0.00 |  |  |
|  | Concerts | 0.00 | 0.00 | 0.00 |  |  |
|  | Mall | 0.00 | 0.00 | 0.00 |  |  |
|  | Media Use | 0.00 | 0.00 | 0.00 |  |  |
|  | Dating | 0.00 | 0.00 | 0.00 |  |  |
|  | Alone Time | 0.00 | 0.00 | 0.00 |  |  |
|  | Facilitating Activities | 0.00 | 0.00 | 0.00 |  |  |
|  | Boredom | 0.00 | 0.00 | 0.00 |  |  |
| Variances: |  |  |  |  |  |  |
|  | S1 | 0.76 | 0.02 | 46.53 | < .001 | 0.90 |
|  | S2 | 0.35 | 0.02 | 21.62 | < .001 | 0.33 |
|  | S3 | 0.53 | 0.02 | 30.65 | < .001 | 0.45 |
|  | S4 | 0.63 | 0.02 | 41.56 | < .001 | 0.68 |
|  | E1 | 0.66 | 0.08 | 8.27 | < .001 | 0.22 |
|  | E2 | 1.14 | 0.06 | 20.15 | < .001 | 0.42 |
|  | V1 | 0.00 | 0.00 | 0.00 |  |  |
|  | J1 | 0.00 | 0.00 | 0.00 |  |  |
|  | CO1 | 0.00 | 0.00 | 0.00 |  |  |
|  | CM1 | 0.00 | 0.00 | 0.00 |  |  |
|  | M1 | 1.06 | 0.05 | 21.46 | < .001 | 0.41 |
|  | M2 | 0.82 | 0.06 | 14.48 | < .001 | 0.31 |
|  | M3 | 2.41 | 0.05 | 46.39 | < .001 | 0.89 |
|  | D1 | 0.00 | 0.00 | 0.00 |  |  |
|  | AL1 | 0.00 | 0.00 | 0.00 |  |  |
|  | C1 | 0.83 | 0.02 | 36.42 | < .001 | 0.55 |
|  | C2 | 0.69 | 0.02 | 38.74 | < .001 | 0.60 |
|  | C3 | 1.27 | 0.03 | 38.86 | < .001 | 0.63 |
|  | C4 | 0.92 | 0.02 | 40.35 | < .001 | 0.87 |
|  | C5 | 0.42 | 0.01 | 45.83 | < .001 | 0.87 |
|  | C6 | 1.96 | 0.05 | 42.52 | < .001 | 0.72 |
|  | B1 | 0.32 | 0.02 | 17.79 | < .001 | 0.19 |
|  | B2 | 0.48 | 0.02 | 24.42 | < .001 | 0.26 |
|  | Enjoyment from School | 1.00 | 1.00 | 1.00 |  |  |
|  | Exercise | 1.00 | 1.00 | 1.00 |  |  |
|  | Volunteer | 1.00 | 1.00 | 1.00 |  |  |
|  | Employment | 1.00 | 1.00 | 1.00 |  |  |
|  | Concerts | 1.00 | 1.00 | 1.00 |  |  |
|  | Mall | 1.00 | 1.00 | 1.00 |  |  |
|  | Media Use | 1.00 | 1.00 | 1.00 |  |  |
|  | Dating | 1.00 | 1.00 | 1.00 |  |  |
|  | Alone Time | 1.00 | 1.00 | 1.00 |  |  |
|  | Facilitating Activities | 1.00 | 1.00 | 1.00 |  |  |
|  | Boredom | 1.00 | 1.00 | 1.00 |  |  |
| * Tau equivalence assumed | | | | | | |

| **Supplemental Table S6** | | | | | | |
| --- | --- | --- | --- | --- | --- | --- |
| *Summary of results for Alcohol Subsample Measurement Model (Model 2)* | | | | | | |
|  |  | *Unstandardized B* | *S.E.* | *z* | *p* | *Standardized β* |
| Latent Variables: | |  |  |  |  |  |
| Enjoyment from School | |  |  |  |  |  |
|  | S1 | 0.30 | 0.02 | 13.13 | < .001 | 0.32 |
|  | S2 | 0.83 | 0.02 | 37.13 | < .001 | 0.81 |
|  | S3 | 0.79 | 0.02 | 34.28 | < .001 | 0.75 |
|  | S4 | 0.52 | 0.02 | 23.53 | < .001 | 0.54 |
| Exercise | |  |  |  |  |  |
|  | E1 | 1.59 | 0.05 | 32.72 | < .001 | 0.91 |
|  | E2 | 1.21 | 0.04 | 27.50 | < .001 | 0.74 |
| Volunteer | |  |  |  |  |  |
|  | V1 | 1.06 | 0.02 | 64.54 | < .001 | 1.00 |
| Employment | |  |  |  |  |  |
|  | J1 | 1.59 | 0.03 | 64.53 | < .001 | 1.00 |
| Going to Concerts | |  |  |  |  |  |
|  | CO1 | 0.63 | 0.01 | 64.62 | < .001 | 1.00 |
| Going to the Mall | |  |  |  |  |  |
|  | MA1 | 0.85 | 0.01 | 64.50 | < .001 | 1.00 |
| Media Use | |  |  |  |  |  |
|  | M1 | 1.17 | 0.04 | 30.38 | < .001 | 0.76 |
|  | M2 | 1.38 | 0.04 | 32.88 | < .001 | 0.85 |
|  | M3 | 0.60 | 0.04 | 15.11 | < .001 | 0.36 |
| Dating | |  |  |  |  |  |
|  | D1 | 1.36 | 0.02 | 63.66 | < .001 | 1.00 |
| Alone Time | |  |  |  |  |  |
|  | AL1 | 1.17 | 0.02 | 64.58 | < .001 | 1.00 |
| Facilitating Activities | |  |  |  |  |  |
|  | C1 | 0.78 | 0.03 | 29.69 | < .001 | 0.66 |
|  | C2 | 0.72 | 0.02 | 30.39 | < .001 | 0.67 |
|  | C3 | 0.87 | 0.03 | 26.59 | < .001 | 0.61 |
|  | C4 | 0.33 | 0.03 | 12.11 | < .001 | 0.33 |
|  | C5 | 0.21 | 0.02 | 12.69 | < .001 | 0.31 |
|  | C6 | 0.77 | 0.04 | 21.45 | < .001 | 0.50 |
| Boredom | |  |  |  |  |  |
|  | B1* | 1.17 | 0.02 | 52.45 | < .001 | 0.90 |
|  | B2* | 1.17 | 0.02 | 52.45 | < .001 | 0.87 |
| Alcohol Involved | |  |  |  |  |  |
|  | AI1 | 0.42 | 0.02 | 19.03 | < .001 | 0.45 |
|  | AI2 | 0.68 | 0.02 | 30.32 | < .001 | 0.67 |
|  | AI3 | 0.42 | 0.01 | 30.09 | < .001 | 0.66 |
|  | AI4 | 0.25 | 0.01 | 21.25 | < .001 | 0.50 |
|  | AI5 | 0.33 | 0.01 | 28.90 | < .001 | 0.64 |
|  | AI6 | 0.56 | 0.02 | 36.78 | < .001 | 0.76 |
|  | AI7 | 0.75 | 0.02 | 32.45 | < .001 | 0.70 |
|  | AI8 | 0.53 | 0.02 | 30.80 | < .001 | 0.66 |
| Alcohol Use | |  |  |  |  |  |
|  | A1 | 1.20 | 0.03 | 37.76 | < .001 | 0.77 |
|  | A2 | 1.37 | 0.03 | 49.40 | < .001 | 0.90 |
|  | A3 | 0.85 | 0.02 | 41.71 | < .001 | 0.80 |
|  | A4 | 0.56 | 0.02 | 30.82 | < .001 | 0.65 |
| Covariances: | |  |  |  |  |  |
| AI4 | |  |  |  |  |  |
|  | AI5 | 0.09 | 0.01 | 18.26 | < .001 | 0.54 |
| AI2 | |  |  |  |  |  |
|  | AI7 | 0.23 | 0.02 | 12.49 | < .001 | 0.40 |
| A3 | |  |  |  |  |  |
|  | A4 | 0.20 | 0.01 | 14.99 | < .001 | 0.47 |
| Enjoyment from School | |  |  |  |  |  |
|  | Exercise | 0.25 | 0.03 | 9.35 | < .001 | 0.25 |
|  | Volunteer | 0.23 | 0.02 | 9.72 | < .001 | 0.23 |
|  | Employment | -0.08 | 0.03 | -3.29 | < .001 | -0.08 |
|  | Concerts | 0.02 | 0.03 | 0.74 | 0.46 | 0.02 |
|  | Mall | 0.10 | 0.03 | 3.92 | < .001 | 0.10 |
|  | Media Use | -0.18 | 0.03 | -6.61 | < .001 | -0.18 |
|  | Dating | 0.01 | 0.03 | 0.29 | 0.77 | 0.01 |
|  | Alone Time | 0.01 | 0.03 | 0.38 | 0.70 | 0.01 |
|  | Facilitating Activities | 0.09 | 0.03 | 3.02 | < .001 | 0.09 |
|  | Boredom | -0.28 | 0.03 | -10.48 | < .001 | -0.28 |
|  | Alcohol Involved | -0.17 | 0.03 | -6.19 | < .001 | -0.17 |
|  | Alcohol Use | -0.16 | 0.03 | -5.96 | < .001 | -0.16 |
| Exercise | |  |  |  |  |  |
|  | Volunteer | 0.23 | 0.02 | 10.19 | < .001 | 0.23 |
|  | Employment | -0.08 | 0.02 | -3.17 | < .001 | -0.08 |
|  | Concerts | 0.09 | 0.02 | 3.98 | < .001 | 0.09 |
|  | Mall | 0.10 | 0.02 | 4.16 | < .001 | 0.10 |
|  | Media Use | -0.16 | 0.03 | -6.08 | < .001 | -0.16 |
|  | Dating | 0.19 | 0.02 | 8.22 | < .001 | 0.19 |
|  | Alone Time | -0.03 | 0.02 | -1.05 | 0.29 | -0.03 |
|  | Facilitating Activities | 0.34 | 0.03 | 13.23 | < .001 | 0.34 |
|  | Boredom | -0.31 | 0.03 | -12.27 | < .001 | -0.31 |
|  | Alcohol Involved | 0.05 | 0.03 | 1.93 | 0.05 | 0.05 |
|  | Alcohol Use | 0.09 | 0.03 | 3.50 | < .001 | 0.09 |
| Volunteer | |  |  |  |  |  |
|  | Employment | 0.02 | 0.02 | 1.04 | 0.30 | 0.02 |
|  | Concerts | 0.18 | 0.02 | 8.59 | < .001 | 0.18 |
|  | Mall | 0.11 | 0.02 | 5.15 | < .001 | 0.11 |
|  | Media Use | -0.12 | 0.02 | -4.87 | < .001 | -0.12 |
|  | Dating | 0.09 | 0.02 | 3.88 | < .001 | 0.09 |
|  | Alone Time | 0.01 | 0.02 | 0.39 | 0.70 | 0.01 |
|  | Facilitating Activities | 0.18 | 0.03 | 7.02 | < .001 | 0.18 |
|  | Boredom | -0.11 | 0.03 | -4.44 | < .001 | -0.11 |
|  | Alcohol Involved | 0.00 | 0.03 | -0.09 | 0.93 | 0.00 |
|  | Alcohol Use | -0.02 | 0.02 | -0.83 | 0.41 | -0.02 |
| Employment | |  |  |  |  |  |
|  | Concerts | 0.05 | 0.02 | 2.28 | 0.02 | 0.05 |
|  | Mall | 0.03 | 0.02 | 1.39 | 0.16 | 0.03 |
|  | Media Use | 0.03 | 0.03 | 1.29 | 0.20 | 0.03 |
|  | Dating | 0.10 | 0.02 | 4.50 | < .001 | 0.10 |
|  | Alone Time | -0.10 | 0.02 | -4.37 | < .001 | -0.10 |
|  | Facilitating Activities | 0.10 | 0.03 | 3.87 | < .001 | 0.10 |
|  | Boredom | -0.10 | 0.03 | -3.99 | < .001 | -0.10 |
|  | Alcohol Involved | 0.05 | 0.03 | 1.98 | 0.05 | 0.05 |
|  | Alcohol Use | 0.09 | 0.02 | 3.68 | < .001 | 0.09 |
| Concerts | |  |  |  |  |  |
|  | Mall | 0.22 | 0.02 | 10.53 | < .001 | 0.22 |
|  | Media Use | 0.00 | 0.03 | 0.07 | 0.94 | 0.00 |
|  | Dating | 0.14 | 0.02 | 6.21 | < .001 | 0.14 |
|  | Alone Time | 0.03 | 0.02 | 1.12 | 0.26 | 0.03 |
|  | Facilitating Activities | 0.34 | 0.02 | 14.31 | < .001 | 0.34 |
|  | Boredom | -0.12 | 0.03 | -5.05 | < .001 | -0.12 |
|  | Alcohol Involved | 0.17 | 0.02 | 7.23 | < .001 | 0.17 |
|  | Alcohol Use | 0.15 | 0.02 | 6.37 | < .001 | 0.15 |
| Mall | |  |  |  |  |  |
|  | Media Use | 0.23 | 0.02 | 9.61 | < .001 | 0.23 |
|  | Dating | 0.16 | 0.02 | 7.26 | < .001 | 0.16 |
|  | Alone Time | 0.02 | 0.02 | 0.79 | 0.43 | 0.02 |
|  | Facilitating Activities | 0.43 | 0.02 | 19.43 | < .001 | 0.43 |
|  | Boredom | -0.06 | 0.03 | -2.40 | 0.02 | -0.06 |
|  | Alcohol Involved | 0.09 | 0.02 | 3.83 | < .001 | 0.09 |
|  | Alcohol Use | 0.05 | 0.02 | 2.32 | 0.02 | 0.05 |
| Media Use | |  |  |  |  |  |
|  | Dating | 0.11 | 0.03 | 4.49 | < .001 | 0.11 |
|  | Alone Time | 0.02 | 0.03 | 0.86 | 0.39 | 0.02 |
|  | Facilitating Activities | 0.16 | 0.03 | 5.73 | < .001 | 0.16 |
|  | Boredom | 0.18 | 0.03 | 6.41 | < .001 | 0.18 |
|  | Alcohol Involved | 0.16 | 0.03 | 5.77 | < .001 | 0.16 |
|  | Alcohol Use | 0.10 | 0.03 | 3.80 | < .001 | 0.10 |
| Dating | |  |  |  |  |  |
|  | Alone Time | -0.04 | 0.02 | -1.56 | 0.12 | -0.04 |
|  | Facilitating Activities | 0.40 | 0.02 | 17.91 | < .001 | 0.40 |
|  | Boredom | -0.16 | 0.02 | -6.67 | < .001 | -0.16 |
|  | Alcohol Involved | 0.17 | 0.02 | 6.84 | < .001 | 0.17 |
|  | Alcohol Use | 0.14 | 0.02 | 6.04 | < .001 | 0.14 |
| Alone Time | |  |  |  |  |  |
|  | Facilitating Activities | 0.07 | 0.03 | 2.69 | 0.01 | 0.07 |
|  | Boredom | 0.08 | 0.03 | 3.08 | < .001 | 0.08 |
|  | Alcohol Involved | 0.02 | 0.03 | 0.62 | 0.53 | 0.02 |
|  | Alcohol Use | -0.02 | 0.02 | -0.82 | 0.41 | -0.02 |
| Facilitating Activities | |  |  |  |  |  |
|  | Boredom | -0.28 | 0.03 | -10.10 | < .001 | -0.28 |
|  | Alcohol Involved | 0.39 | 0.03 | 14.80 | < .001 | 0.39 |
|  | Alcohol Use | 0.40 | 0.02 | 16.46 | < .001 | 0.40 |
| Boredom | |  |  |  |  |  |
|  | Alcohol Involved | 0.01 | 0.03 | 0.26 | 0.80 | 0.01 |
|  | Alcohol Use | -0.08 | 0.03 | -3.16 | < .001 | -0.08 |
| Alcohol Involved | |  |  |  |  |  |
|  | Alcohol Use | 0.75 | 0.02 | 48.87 | < .001 | 0.75 |
| Intercepts: |  |  |  |  |  |  |
|  | S1 | 4.00 | 0.02 | 195.53 | < .001 | 4.28 |
|  | S2 | 2.94 | 0.02 | 131.69 | < .001 | 2.88 |
|  | S3 | 2.65 | 0.02 | 115.13 | < .001 | 2.52 |
|  | S4 | 2.59 | 0.02 | 123.64 | < .001 | 2.71 |
|  | E1 | 4.28 | 0.04 | 112.00 | < .001 | 2.45 |
|  | E2 | 3.97 | 0.04 | 105.58 | < .001 | 2.41 |
|  | V1 | 2.24 | 0.02 | 96.14 | < .001 | 2.11 |
|  | J1 | 1.86 | 0.04 | 53.22 | < .001 | 1.17 |
|  | CO1 | 1.56 | 0.01 | 112.85 | < .001 | 2.47 |
|  | CM1 | 2.77 | 0.02 | 148.97 | < .001 | 3.26 |
|  | M1 | 4.14 | 0.03 | 122.17 | < .001 | 2.67 |
|  | M2 | 3.84 | 0.04 | 107.20 | < .001 | 2.35 |
|  | M3 | 3.71 | 0.04 | 102.22 | < .001 | 2.24 |
|  | D1 | 2.16 | 0.03 | 71.43 | < .001 | 1.58 |
|  | AL1 | 5.09 | 0.03 | 198.60 | < .001 | 4.35 |
|  | C1 | 4.18 | 0.03 | 162.10 | < .001 | 3.54 |
|  | C2 | 2.98 | 0.02 | 127.37 | < .001 | 2.79 |
|  | C3 | 2.76 | 0.03 | 86.76 | < .001 | 1.92 |
|  | C4 | 2.81 | 0.02 | 114.98 | < .001 | 2.80 |
|  | C5 | 2.47 | 0.02 | 168.30 | < .001 | 3.68 |
|  | C6 | 3.70 | 0.03 | 108.79 | < .001 | 2.38 |
|  | B1 | 3.46 | 0.03 | 113.80 | < .001 | 2.65 |
|  | B2 | 3.30 | 0.03 | 105.38 | < .001 | 2.45 |
|  | AI1 | 1.86 | 0.02 | 86.69 | < .001 | 1.98 |
|  | AI2 | 1.99 | 0.02 | 86.16 | < .001 | 1.95 |
|  | AI3 | 1.25 | 0.01 | 87.47 | < .001 | 1.98 |
|  | AI4 | 1.14 | 0.01 | 101.98 | < .001 | 2.31 |
|  | AI5 | 1.14 | 0.01 | 100.02 | < .001 | 2.26 |
|  | AI6 | 1.33 | 0.02 | 80.46 | < .001 | 1.81 |
|  | AI7 | 1.98 | 0.02 | 81.80 | < .001 | 1.85 |
|  | AI8 | 1.37 | 0.02 | 75.93 | < .001 | 1.71 |
|  | A1 | 2.38 | 0.04 | 68.18 | < .001 | 1.52 |
|  | A2 | 2.99 | 0.03 | 90.10 | < .001 | 1.98 |
|  | A3 | 1.70 | 0.02 | 72.35 | < .001 | 1.59 |
|  | A4 | 1.38 | 0.02 | 73.57 | < .001 | 1.63 |
|  | Enjoyment from School | 0.00 | 0.00 | 0.00 |  |  |
|  | Exercise | 0.00 | 0.00 | 0.00 |  |  |
|  | Volunteer | 0.00 | 0.00 | 0.00 |  |  |
|  | Employment | 0.00 | 0.00 | 0.00 |  |  |
|  | Concerts | 0.00 | 0.00 | 0.00 |  |  |
|  | Mall | 0.00 | 0.00 | 0.00 |  |  |
|  | Media Use | 0.00 | 0.00 | 0.00 |  |  |
|  | Dating | 0.00 | 0.00 | 0.00 |  |  |
|  | Alone Time | 0.00 | 0.00 | 0.00 |  |  |
|  | Facilitating Activities | 0.00 | 0.00 | 0.00 |  |  |
|  | Boredom | 0.00 | 0.00 | 0.00 |  |  |
|  | Alcohol Involved | 0.00 | 0.00 | 0.00 |  |  |
|  | Alcohol Use | 0.00 | 0.00 | 0.00 |  |  |
| Variances: |  |  |  |  |  |  |
|  | S1 | 0.79 | 0.03 | 31.31 | < .001 | 0.90 |
|  | S2 | 0.36 | 0.02 | 15.14 | < .001 | 0.34 |
|  | S3 | 0.49 | 0.02 | 20.22 | < .001 | 0.44 |
|  | S4 | 0.65 | 0.02 | 28.49 | < .001 | 0.71 |
|  | E1 | 0.53 | 0.13 | 4.22 | < .001 | 0.17 |
|  | E2 | 1.25 | 0.08 | 15.06 | < .001 | 0.46 |
|  | V1 | 0.00 | 0.00 | 0.00 |  |  |
|  | J1 | 0.00 | 0.00 | 0.00 |  |  |
|  | CO1 | 0.00 | 0.00 | 0.00 |  |  |
|  | CM1 | 0.00 | 0.00 | 0.00 |  |  |
|  | M1 | 1.02 | 0.07 | 14.89 | < .001 | 0.43 |
|  | M2 | 0.76 | 0.09 | 8.67 | < .001 | 0.29 |
|  | M3 | 2.40 | 0.08 | 31.06 | < .001 | 0.87 |
|  | D1 | 0.00 | 0.00 | 0.00 |  |  |
|  | AL1 | 0.00 | 0.00 | 0.00 |  |  |
|  | C1 | 0.79 | 0.03 | 24.91 | < .001 | 0.56 |
|  | C2 | 0.63 | 0.03 | 24.49 | < .001 | 0.55 |
|  | C3 | 1.30 | 0.05 | 26.30 | < .001 | 0.63 |
|  | C4 | 0.90 | 0.03 | 27.83 | < .001 | 0.89 |
|  | C5 | 0.41 | 0.01 | 31.31 | < .001 | 0.91 |
|  | C6 | 1.82 | 0.06 | 29.30 | < .001 | 0.75 |
|  | B1 | 0.34 | 0.03 | 12.85 | < .001 | 0.20 |
|  | B2 | 0.45 | 0.03 | 15.82 | < .001 | 0.25 |
|  | AI1 | 0.71 | 0.02 | 29.48 | < .001 | 0.80 |
|  | AI2 | 0.58 | 0.02 | 25.71 | < .001 | 0.56 |
|  | AI3 | 0.23 | 0.01 | 26.75 | < .001 | 0.57 |
|  | AI4 | 0.18 | 0.01 | 28.87 | < .001 | 0.75 |
|  | AI5 | 0.15 | 0.01 | 26.68 | < .001 | 0.59 |
|  | AI6 | 0.22 | 0.01 | 23.00 | < .001 | 0.42 |
|  | AI7 | 0.58 | 0.02 | 24.65 | < .001 | 0.51 |
|  | AI8 | 0.36 | 0.01 | 26.91 | < .001 | 0.56 |
|  | A1 | 1.00 | 0.04 | 24.54 | < .001 | 0.41 |
|  | A2 | 0.43 | 0.03 | 13.65 | < .001 | 0.19 |
|  | A3 | 0.42 | 0.02 | 24.81 | < .001 | 0.37 |
|  | A4 | 0.41 | 0.02 | 27.49 | < .001 | 0.57 |
|  | Enjoyment from School | 1.00 | 1.00 | 1.00 |  |  |
|  | Exercise | 1.00 | 1.00 | 1.00 |  |  |
|  | Volunteer | 1.00 | 1.00 | 1.00 |  |  |
|  | Employment | 1.00 | 1.00 | 1.00 |  |  |
|  | Concerts | 1.00 | 1.00 | 1.00 |  |  |
|  | Mall | 1.00 | 1.00 | 1.00 |  |  |
|  | Media Use | 1.00 | 1.00 | 1.00 |  |  |
|  | Dating | 1.00 | 1.00 | 1.00 |  |  |
|  | Alone Time | 1.00 | 1.00 | 1.00 |  |  |
|  | Facilitating Activities | 1.00 | 1.00 | 1.00 |  |  |
|  | Boredom | 1.00 | 1.00 | 1.00 |  |  |
|  | Alcohol Involved | 1.00 | 1.00 | 1.00 |  |  |
|  | Alcohol Use | 1.00 | 1.00 | 1.00 |  |  |
| * Tau equivalence assumed | | | | | | |
